# Advancing interactive evidence maps: Visualising service commissioning options alongside research

**DOI:** 10.1002/cesm.70003

**Published:** 2024-10-05

**Authors:** Helen Burchett, Claire Stansfield, Wendy Macdowall, Michelle Richardson, Samantha Dick, Kelly Dickson, Preethy D'Souza, Claire Khouja, Irene Kwan, Gary Raine, Amanda Sowden, Katy Sutcliffe, James Thomas

**Affiliations:** ^1^ Faculty of Public Health Policy London School of Hygiene & Tropical Medicine London UK; ^2^ EPPI Centre, UCL Social Research Institute University College London London UK; ^3^ School of Public Health University College Cork Cork Ireland; ^4^ Centre for Reviews & Dissemination University of York York UK

**Keywords:** evidence map, evidence synthesis, gap map

## Abstract

**Background:**

Interactive evidence maps typically visualise characteristics of research evidence, and gaps in evidence, in a particular field.

**Aims, Materials & Methods:**

Here we present an example of an evidence map on digital drug and alcohol interventions in which the research evidence is supplemented with information about interventions in use (or available for use) in England. We used systematic review methods to identify systematic reviews of intervention effectiveness and an online survey to identify interventions in England.

**Results:**

Eighteen reviews and 40 interventions were included in the online map.

**Discussion & Conclusion:**

To our knowledge, this is the first map to juxtapose research and practice in this way. By extending evidence maps to include data on service provision, it becomes easier to see whether research and practice are aligned and where gaps in either evidence or practice (or both) exist.

## 1 BACKGROUND

Given the proliferation of research publications in recent decades, it is now accepted that identifying research in more than an ad‐hoc manner requires time and specialist searching skills, along with access to material that may be behind a journal's pay‐wall [1, 2, 3]. It is therefore understandable that decision‐makers and other evidence users often find it difficult to identify what research exists and where research gaps remain.

Evidence maps visually represent research evidence in a particular field or topic [1]. Although grounded in systematic techniques, they typically have a broader scope than systematic reviews as they aim to present all available research in a given field, rather than synthesising study findings to answer a specific, often narrower, question. Such visual representations of available research have been recognised as important and desirable by those involved in evidence‐informed decision‐making [4].

However, to inform decision‐making in specific contexts, it is necessary to not only be familiar with the research evidence, but to be aware of what has been implemented in practice and to understand how the two relate.

Here, we report a case study of how we produced an evidence map for Public Health England (PHE) presenting review‐level evidence of digital drug and alcohol intervention effectiveness alongside descriptions of the interventions that were in use in the English context. Further details about the methods, and results, can be found in the full project report [5].

## 2 METHODS

### 2.1 Identifying effectiveness evidence

To identify intervention effectiveness evidence, we conducted a systematic search of 29 bibliographic databases and registries. Following duplicate removal, titles and abstracts were screened against pre‐specified inclusion criteria, using a priority screening approach in EPPI‐Reviewer [6]. More details about the priority screening process are provided in the full report but, in summary, it uses machine learning based on text mining to prioritise the most likely relevant references. This means that those most likely to be included are manually screened first, prioritising them to speed up the screening process [7]. Manual screening was stopped once an appropriate cut off point was determined, when it was believed that we had identified all, or almost all, of the relevant references. The full texts of systematic reviews were retrieved and screened for inclusion.

Included systematic reviews were quality appraised using AMSTAR 2 [8] and coded according to pre‐defined characteristics, including the interventions' focus (e.g. prevention and early intervention, treatment and recovery, or sustaining recovery) and which intervention components were employed. Summary findings of effectiveness from meta‐analyses (but not narrative syntheses) were also extracted.

### 2.2 Identifying interventions in England

We conducted an online survey among those involved in developing, commissioning, prescribing, recommending or evaluating digital interventions for alcohol or drug use. We asked participants to identify and provide information about digital interventions that were either in use, or potentially available for use, in England. We shared the online survey link widely, as well as identifying known interventions from personal communication with PHE, advisory group members, and intervention developers. Once we had identified interventions through the survey and personal communication, we produced a structured summary description for each intervention. The information for these summaries was taken not only from survey responses; we also reviewed the interventions themselves (i.e. the app or website), and publicly available descriptions of them where available. As with the included systematic reviews, interventions were coded according to whether they focused on prevention, treatment, or recovery and which intervention components they employed. Descriptions and codes were checked for accuracy with intervention developers.

### 2.3 Producing interactive, visual maps

The coding and descriptive summaries were managed within EPPI‐Reviewer [9] and a visual map was then created using EPPI‐Mapper software (v1.2.0) [10]. Since the appearance and functionality of the map are fundamental to its appeal and use, these were developed iteratively following feedback from stakeholders. An organising framework was developed using select codes: the intervention focus and components provided the *X* axis and whether the intervention targeted alcohol, drugs or both formed the *Y* axis. Succinct headings help make the map clear to use, and they include a glossary and a section explaining how to use the map. More information about developing the maps can be found in our blog post [11]. Technical support was provided by the EPPI‐Reviewer support team.

## 3 FINDINGS

**Figure 1:**
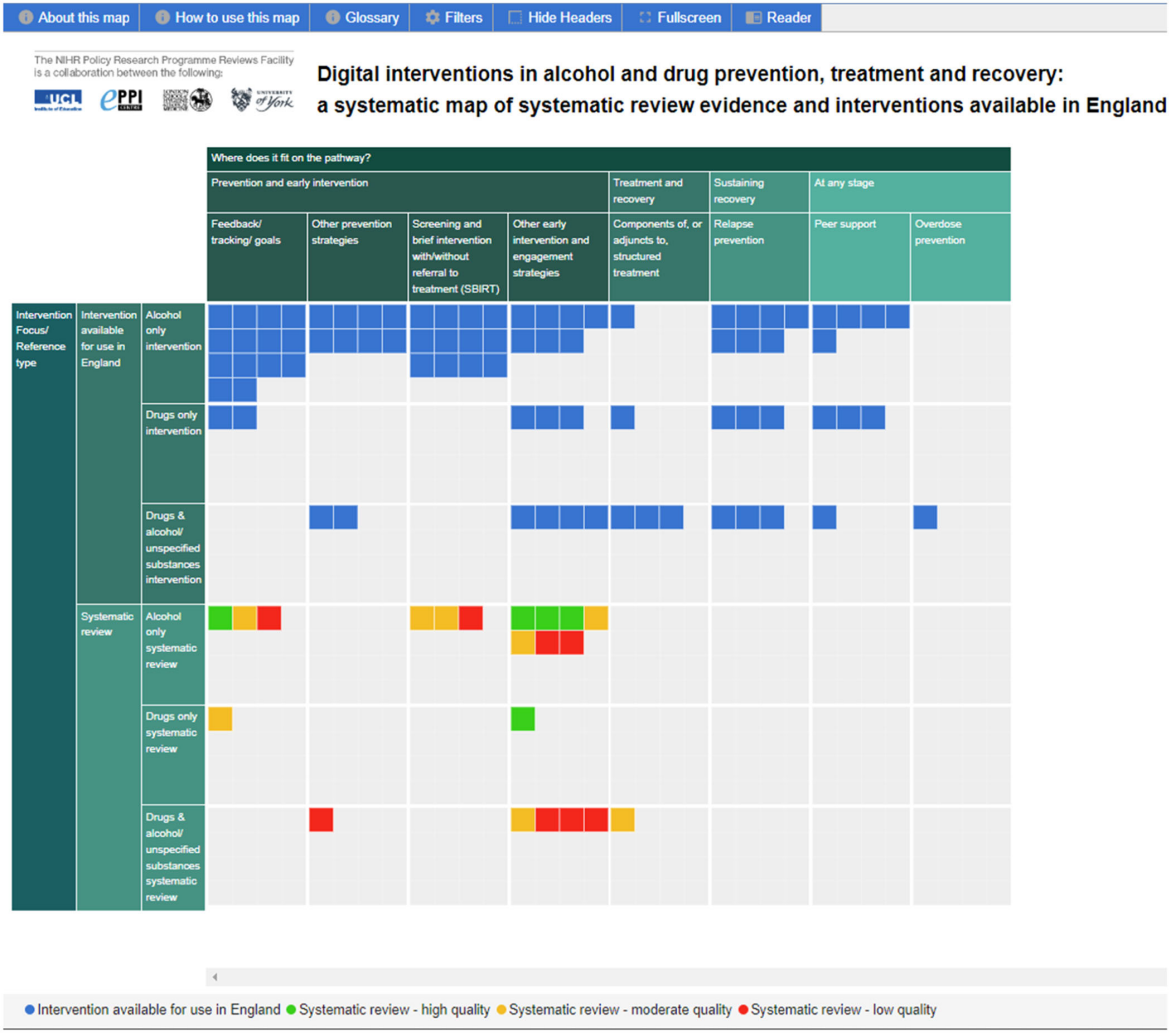
Evidence map of systematic reviews and interventions available in England.

The searches identified 20,961 references after duplicate removal and 14,402 were screened on title and abstract. The remaining references were excluded based on priority screening. The full texts of 87 systematic reviews were screened, resulting in the inclusion of 18 systematic reviews in the map (see Figure 1).

Fifty‐nine people responded to the survey, resulting in the inclusion of 23 unique interventions (since some were reported by more than one person and others were excluded for being out of scope). A further 17 interventions were identified through personal communications, resulting in a total of 40 interventions being included in the map (see Figure 1).

**Figure 2:**
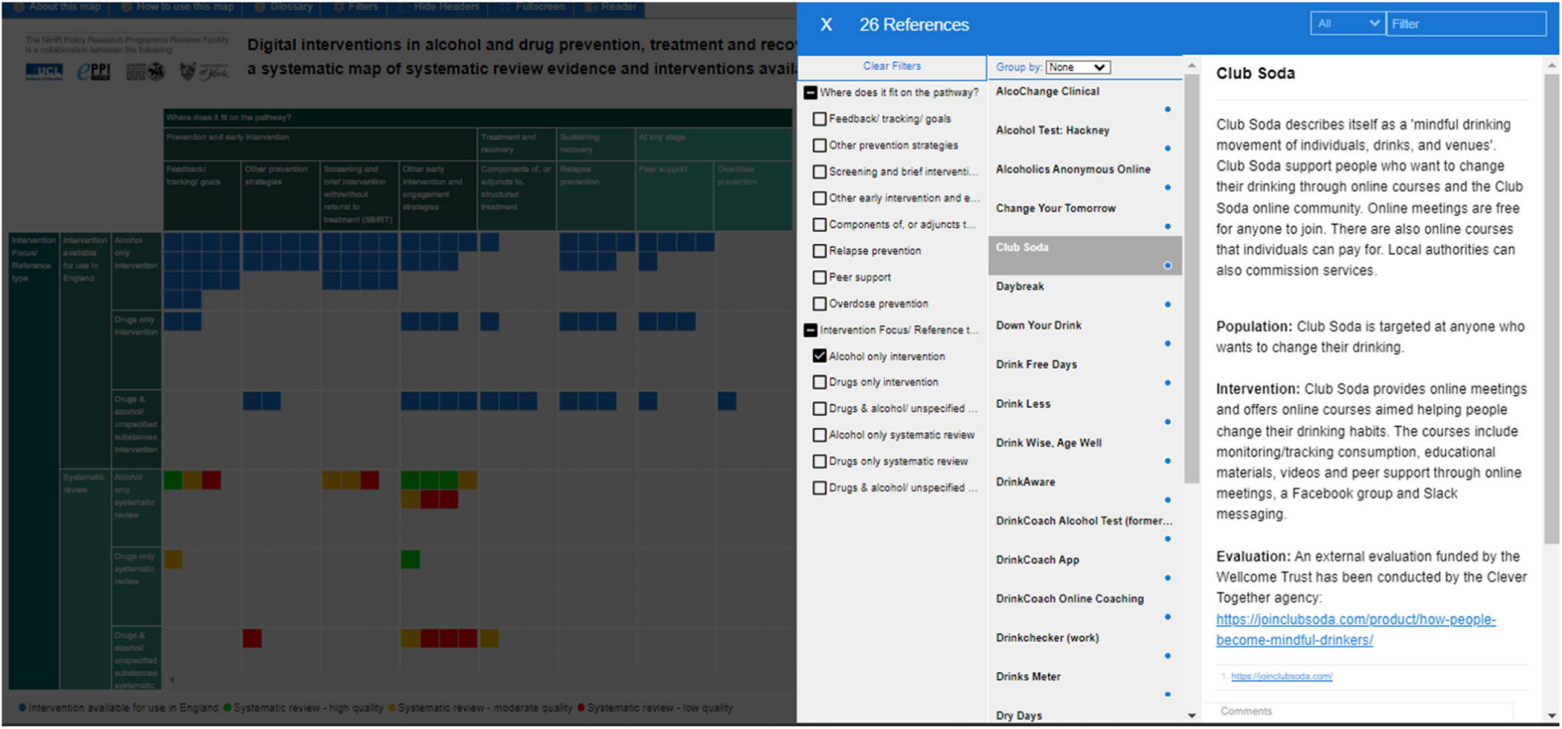
Example of filtering records to show the list of “feedback/tracking/goals” interventions for “alcohol only interventions,” highlighting the detail for one intervention.

The map is available online and offers different options for exploring the data and focusing on details within it. It can be browsed, searched using free‐text, or filtered, either using the “filters” heading, or by simply clicking on the row, column or cell of interest (e.g., “feedback/tracking/goals” and “alcohol only interventions”) to bring up a list of the interventions with those codes. This then provides a list of the reviews or interventions within the subset, with details provided for each review or intervention that can be read in turn (see Figure 2). Whichever way the map is searched, or when clicking the “reader” heading (to show all entries), users can read detailed descriptions about each intervention or systematic review. The accompanying report contains further details and interpretation that are not in the map [5].

The map shows that the majority of interventions and systematic reviews focused on alcohol rather than drugs, and on prevention and early intervention rather than treatment or recovery. Several intervention strategies were identified for which there were no high‐quality systematic reviews (e.g., relapse prevention, peer support). The map also shows the intervention strategies for which there were neither available interventions nor high‐quality systematic reviews (e.g., screening and brief interventions for drugs or drugs and alcohol).

## 4 DISCUSSION

Interactive evidence maps present data visually, enabling the user to see at‐a‐glance what research exists, or is lacking, across a broad topic, whilst also allowing the user to explore areas in further detail. To our knowledge, our map of available drug and alcohol interventions and systematic reviews of intervention effectiveness is the first to juxtapose both research and practice in a single map.

The benefits of this approach are numerous:

- They provide an at‐a‐glance overview of not only the research evidence for different types of interventions, but also which interventions are currently in, or available, for use.
- Users can assess whether available interventions are underpinned by rigorous research evidence.
- Users can easily identify gaps in the evidence base and in current (or available) service provision.
- By combining international research with national service provision, or commissioning options, the map provides a means of exploring the extent to which research from elsewhere aligns with national service provision contexts.

A clear limitation is the substantial amount of time and funding required to complete maps with this degree of detail.

Evidence maps can be used to identify where attention has been focused within a broad field. For example, in our map we identified more interventions and evidence relating to prevention than to treatment or recovery, and to alcohol than drugs. This can help research and service commissioners, as well as those advocating for increased resources or support for neglected areas.

Maps such as these provide a snapshot of research and service provision; for example our map provides a pre‐COVID “baseline” for digital drug and alcohol research and service commissioning. A subsequent map could use this to contrast the extent to which developments in the field have been made, however there is also an argument for the production of “living” maps, which are continually or frequently updated. Whilst living maps have some obvious benefits, they also have substantial resource implications—which could affect the level of detail provided in the map and the extent to which processes are automated [12, 13].

Now that the threshold of what could be incorporated into evidence maps has been expanded, other aspects could be considered in future maps. For example, needs are an obvious next step to incorporate, as this would allow an assessment of the extent to which existing research and practice align with identified needs of users or other stakeholders (while such a combination has previously been incorporated in systematic reviews, we are not aware of this being done within an evidence map) [14, 15]. Identified research priorities could also be incorporated, such as those developed by the James Lind Alliance (JLA) [16]. Conversely, we believe that evidence maps such as the one presented here could facilitate the process of developing consensus on research priorities.

## 5 CONCLUSION

To our knowledge this is the first example of expanding the scope and potential usefulness of evidence maps to combine research and practice. The benefits of juxtaposing the research evidence on effectiveness with information on currently available interventions (in a specific context) are clear. We hope to see future interactive evidence maps push the boundaries even further.

## AUTHOR CONTRIBUTIONS

**Helen Burchett:** Conceptualisation; supervision; investigation; writing—original draft; reviewing and editing. **Claire Stansfield:** Conceptualisation; investigation; visualisation; writing—original draft; reviewing and editing. **Wendy Macdowall:** Investigation; writing—reviewing and editing. **Michelle Richardson:** investigation; writing—reviewing and editing. **Samantha Dick:** Investigation; writing—reviewing and editing. **Kelly Dickson:** Investigation; writing—reviewing and editing. **Preethy D'Souza:** Investigation; writing—reviewing and editing. **Claire Khouja:** Investigation; writing—reviewing and editing. **Irene Kwan:** Investigation; writing—reviewing and editing. **Gary Raine:** Investigation; writing—reviewing and editing. **Amanda Sowden:** Funding acquisition; writing—reviewing and editing. **Katy Sutcliffe:** Conceptualisation; supervision; funding acquisition; investigation; writing—reviewing and editing. **James Thomas:** funding acquisition; writing—reviewing and editing.

## CONFLICT OF INTEREST STATEMENT

No conflicts of interest declared. Samantha Dick is an employee of Eli Lilly since July 2021. Samantha completed this work independently, and before her employment at Eli Lilly. This work is in no way affiliated with Eli Lilly.

## ETHICS STATEMENT

Ethical approval was granted by LSHTM.

## Data Availability

The data that support the findings of this study are available on request from the corresponding author.
